# BRS1 Function in Facilitating Lateral Root Emergence in *Arabidopsis*

**DOI:** 10.3390/ijms18071549

**Published:** 2017-07-18

**Authors:** Qian Deng, Xue Wang, Dongzhi Zhang, Xiaoming Wang, Cuizhu Feng, Shengbao Xu

**Affiliations:** 1 State Key Laboratory of Crop Stress Biology for Arid Areas, College of Agronomy, Northwest A&F University, Yangling 712100, China; 2014050097@nwsuaf.edu.cn (Q.D.); wx0321@nwsuaf.edu.cn (X.W.); wangxm@nwsuaf.edu.cn (X.W.); 2 Ministry of Education Key Laboratory of Cell Activities and Stress Adaptations, School of Life Sciences, Lanzhou University, Lanzhou 730000, China; zhangdzh09@lzu.edu.cn

**Keywords:** serine carboxypeptidase, lateral root emergence, endodermis, BR signaling

## Abstract

The *BRS1* (BRI1 Suppressor 1) gene encodes a serine carboxypeptidase that plays a critical role in the brassinosteroid signaling pathway. However, its specific biological function remains unclear. In this study, the developmental role of BRS1 was investigated in *Arabidopsis thaliana*. We found that overexpressing BRS1 resulted in significantly more lateral roots in different *Arabidopsis* ecotypes (WS2 and Col-0) and in brassinosteroid mutants (*bri1-5* and *det2-28*). Further research showed that BRS1 facilitates the process whereby lateral root primordia break through the endodermis, cortex, and epidermis. Consistent with this, *BRS1* was found to be highly expressed in the root endodermis and accumulated in the extracellular space around the dome of the lateral root primordia. Taken together, these results highlight the role of BRS1 in the process of lateral root emergence and provide new insight into the role of serine carboxypeptidases in plant root development.

## 1. Introduction

BRS1 (BRI1 Suppressor 1) is a serine carboxypeptidase that was recognized to suppress the phenotypes of the brassinosteroid receptor weak mutant *bri1-5*, which showed shorter primary inflorescence stem and secondary inflorescence branch length, compact rosette, and late flowering time [1]. Serine carboxypeptidases (SCPs) belong to the hydrolase family of serine peptidases [2] and are widely expressed in plant organs. Accumulating data indicate that SCPs are involved in protein turnover [3,4], the autolysis of cellular constituents [5,6], and seed development [7,8,9,10]; however, their specific biological function remains to be fully elucidated.

In *Arabidopsis*, 54 SCP-like genes have been identified [11], and these have been divided into three classes [12,13,14]. *BRS1* belongs to class II and shares high sequence similarity with the other four homologs in this class [1,15]. A knockout mutant of BRS1 was reported to exhibit no obvious phenotype [1]. Interestingly, when the homologs of *BRS1* were overexpressed, three of the five could partially rescue the defects of *bri1-5* [15]. These observations provide evidence that the BRS1 family is functionally redundant in *Arabidopsis*.

BRS1 is a secreted serine carboxypeptidase, and is localized within the extracellular space in *Arabidopsis* [16]. Another SCP—NtSCP1, which is localized within the extracellular space in tobacco—has been shown to play a role in cell elongation [17]. Similarly, the overexpression of *BRS1* remarkably increased the length of hypocotyl and secondary inflorescence branch [1], indicating that BRS1 may also participate in cell shape formation due to its site of localization and hydrolase activity.

Lateral root primordia (LRP) originate from pericycle cells [18,19] and must break through the overlying endodermis, cortex, and epidermis before forming a new lateral root (LR). This process is termed LR emergence, and is critical for determining the LR growth rate and distribution [20]. During this process, the original founder cells firstly undergo several anticlinal divisions in the pericycle, and develop as stage I, and then the cells divide periclinally to reach stage II, consisting of two cell layers, which are still located in the pericycle cell [21,22,23]. With constant anticlinal and periclinal divisions, LRPs push apart cells of the endodermis, cortex, and epidermis to pass through during stage III to VIII, and develop autonomous LR [24,25]. The mature cells of the endodermis, cortex, and epidermis are connected to each other via their cell walls and plasmodesmata, especially endodermal cells for which their connections are reinforced by a surrounding Casparian strip. During the endodermis breakthrough process, a spatial accommodation by neighboring cells is required for lateral root expansion and consequent breakthrough outer cells; therefore, the inter-communication between LRP and outer cells plays a critical role in regulation [26]. In summary, an intriguing question emerges about how the overlaying cells detach from each other to make way for LRP growth [26,27,28,29].

A previous study reported that SHORT HYPOCOTYL 2 (SHY2) regulates the changes in endodermal cell wall properties and facilitates the passage of the LRP across the endodermis [26]. Similarly, primordium initiation proteins LBD29 and LBD18 can promote cell separation for LR emergence by increasing the activity of cell wall remodeling enzymes [20,30,31]. XTR6/XTH23 and EXP17 also control cell wall remodeling, and are dramatically increased in expression during LRP pass through the outer cells [24,29]. These observations indicate that the extracellular space experiences considerable changes prior to LR emergence.

Although the hydrolase function of SCPs has been confirmed in a range of organisms, there are few reports on their developmental function. In this study, we carefully investigated the effects of overexpressing *BRS1* on LRs and found that BRS1 facilitates LR emergence, shedding new light on the process of LR emergence and the role of extracellular-localized SCPs. 

## 2. Results

### 2.1. Overexpression of BRS1 Increases the Number of LRs in Arabidopsis

**Figure 1:**
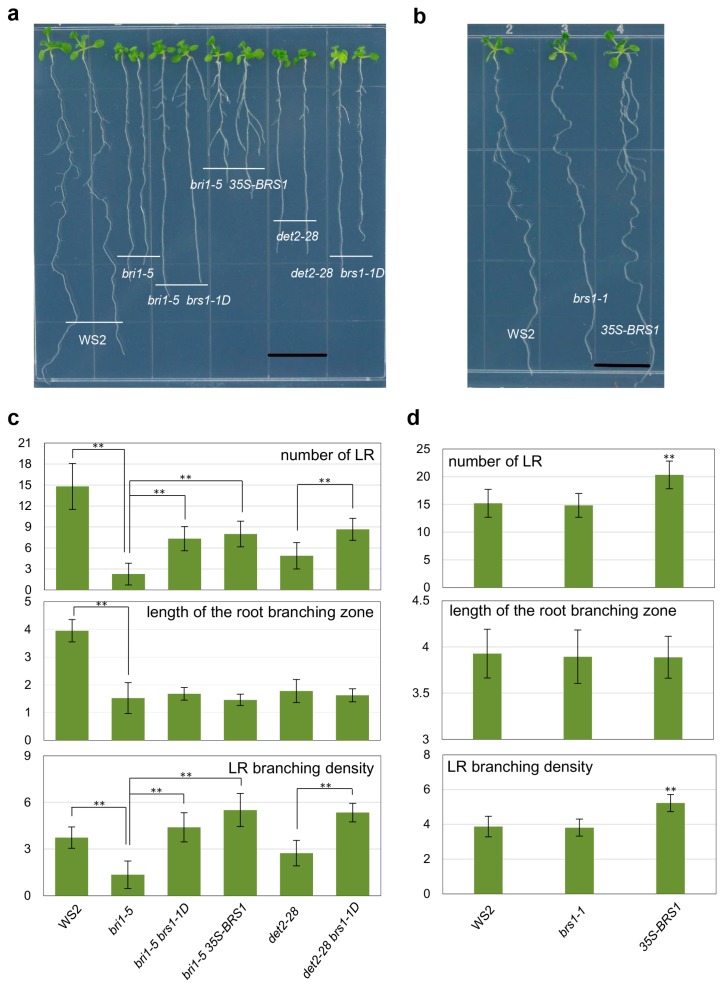
Overexpression of *BRS1* increases the number of lateral root (LRs) in *Arabidopsis*. (**a**) Phenotype comparison of the wild-type plant (WS2) and a range of mutant lines: *bri1-5*, *bri1-5 35S-BRS1*, *bri1-5 brs1-1D*, *det2-28* and *det2-28 brs1-1D*. Imbibed seeds were transferred to half-strength Murashige and Skoog (1/2 MS) medium, and observed at 9 DAG (days after germination). Bar = 1 cm; (**b**) Phenotype comparison of the wild type plant (WS2), the *brs1-1* knockout mutant, and the *35S-BRS1* overexpression line. Imbibed seeds were transferred to 1/2 MS medium and captured photos at 9 DAG. Bar = 1 cm; (**c**) Lateral root number, length of the root branching zone, and lateral root branching density of the wild type plant (WS2), *bri1-5*, *bri1-5 35S-BRS1*, *bri1-5 brs1-1D*, *det2-28* and *det2-28 brs1-1D* were quantified at 9 DAG. Each data bar represents the means ± SE (*n* ≥ 20); (**d**) Lateral root number, length of the root branching zone, and lateral root branching density of the wild type plant (WS2), *35S-BRS1*, and *brs1-1* were quantified at 9 DAG. Each data bar represents the means ± SE (*n* ≥ 20). The asterisks indicate a significant difference from the corresponding control experiment by Student’s *t*-test (** *p* < 0.01).

A previous study showed that length of primary inflorescence stem and secondary inflorescence branch had been heightened, rosettes are bigger rather than curled, and flowering time has been moved up in *bri1-5* by overexpression of *BRS1* [1]. In the work, we found that the LR number and LR branching density—defined as the number of emerged LRs per unit length of the root branching zone [32]—decreased significantly in *bri1-5*, however they dramatically increased in two different *BRS1* overexpression materials *bri1-5 35S-BRS1* and *bri1-5 brs1-1D* (activation tagging line) compared with the *bri1-5* mutant (Figure 1a,c). Interestingly, overexpression of *BRS1* also increased the LR number and LR branching density in the *det2-28* mutant, which is defective in brassinosteroid biosynthesis (Figure 1a,c) [33]. These results indicated that BRS1 is involved in the LR development of brassinosteroid-related mutants.

Further observations demonstrated that the overexpression of *BRS1* also increased the LR number and LR branching density in WT (WS2) plants (Figure 1b,d), suggesting that BRS1 functions in LR development. However, there was no significant difference in the LR number and LR branching density between the *brs1-1* (WS2) mutant and WT (WS2, Figure 1b,d), which was consistent with *BRS1* being a highly redundant gene in *Arabidopsis* [1,15].

### 2.2. Overexpression of BRS1 Promotes LR Emergence

**Figure 2:**
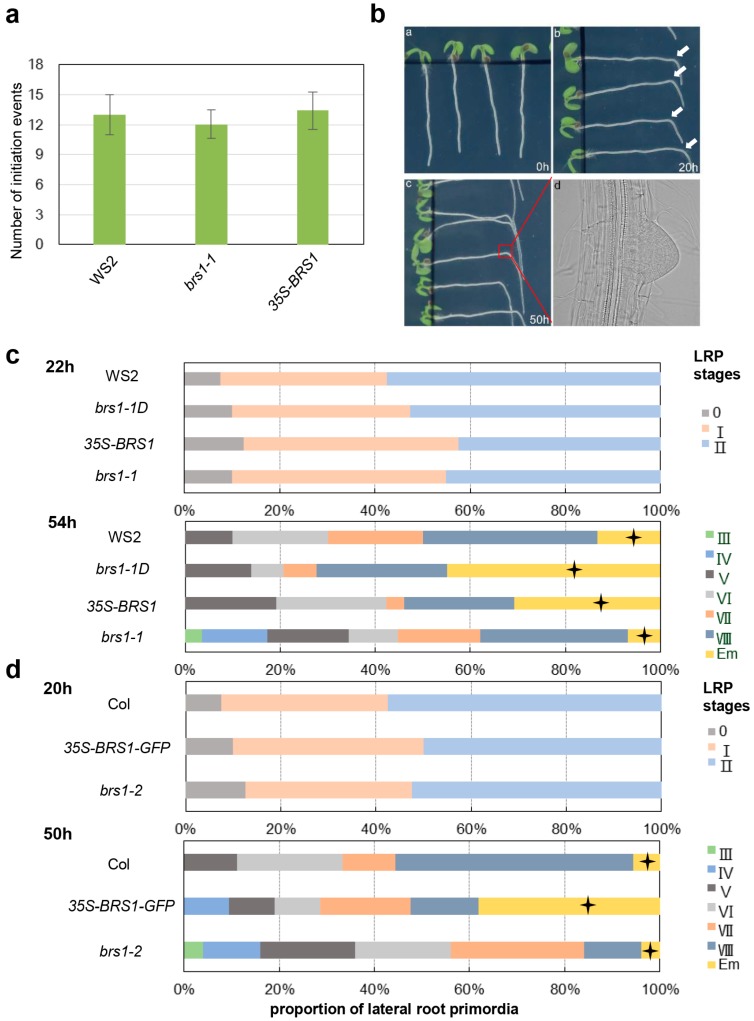
Overexpression of *BRS1* causes accelerated lateral root primordial development. (**a**) The total number of lateral root primordia (LRP) initiation events (which is the sum of LRP and emerged LRs) were calculated at 6 DAG. Each data bar represents the means ± SE (*n* ≥ 20); (**b**) The LRPs were gravistimulated to formation. Imbibed seeds were transferred to 1/2 MS medium without sucrose, and then the 5-day-old wild-type (Col) seedlings were turned to 90° and grew for 20 and 50 h and then the LRPs were observed. Arrowhead shows the bending site, at which LRP initiate synchronously; (**c**,**d**) The lateral root primordia growth rate analysis. (**c**) Imbibed seeds, the wild-type plant WS2, *WS2 brs1-1D*, *35S-BRS1*, and *brs1-1*; (**d**) The wild-type plant Col, *35S-BRS1 GFP* (Col background), and *brs1-2* (Col background) were transferred to 1/2 MS medium and planted by the method mentioned in (**b**), and then the lateral root primordia (LRPs) growth rate were calculated after 22 and 54 h pgi (postgravitropic induction). The lateral root primordia growth rate were measured by the percentage of LRPs at different growth stages (starting from stage I to an emerged lateral root, Em) [21]. Each data bar represents the means ± SE (*n* ≥ 26). The cross star symbol highlighted the proportion of emerged lateral roots.

Next, we questioned whether the increase in the LR number was caused by enhanced LRP initiation. To address this, the number of initiation events in seedlings of *brs1-1D*, *brs1-1*, and WS2 at 6 days after germination (DAG) was counted. No significant difference in the number of initiation events was observed between lines (Figure 2a), indicating that BRS1 may not function in LRP initiation and that the increased number of LRP in BRS1-overexpressed lines may have resulted from the accelerated growth rate of LRP. Therefore, the LRP growth rate was further evaluated using a root bending test (Figure 2b) [34,35,36,37].

In the WT (WS2), LRP were fully induced at the bending point after 22 h gravistimulus treatment, and more than 90% of plants had obvious primordia (including stages I and II; Figure 2c). The induction rate was similar in the Col background, with the mutants *brs1-2* and *35S-BRS1*, and a similar proportion of LRP was observed at stages I and II in these lines after 20 h post-gravitropic induction (Figure 2d), confirming that the initiation and early development of LRP (from stage I to stage II) was not affected by BRS1. However, about 40% of the *35S-BRS1* seedlings showed emerged LRs after 54 h of post-gravitropic induction—a higher proportion than the number of emerged LRs observed in WS2 and *brs1-1* (about 10%), indicating that the overexpression of *BRS1* resulted in faster LR emergence (Figure 2c). The same results were obtained in the Col (Figure 2d) ecotype background, suggesting that BRS1 functions in the acceleration of LR emergence in parental roots, resulting in a visible increase in LR number.

### 2.3. BRS1 Is Highly Expressed in the Root Endodermis

**Figure 3:**
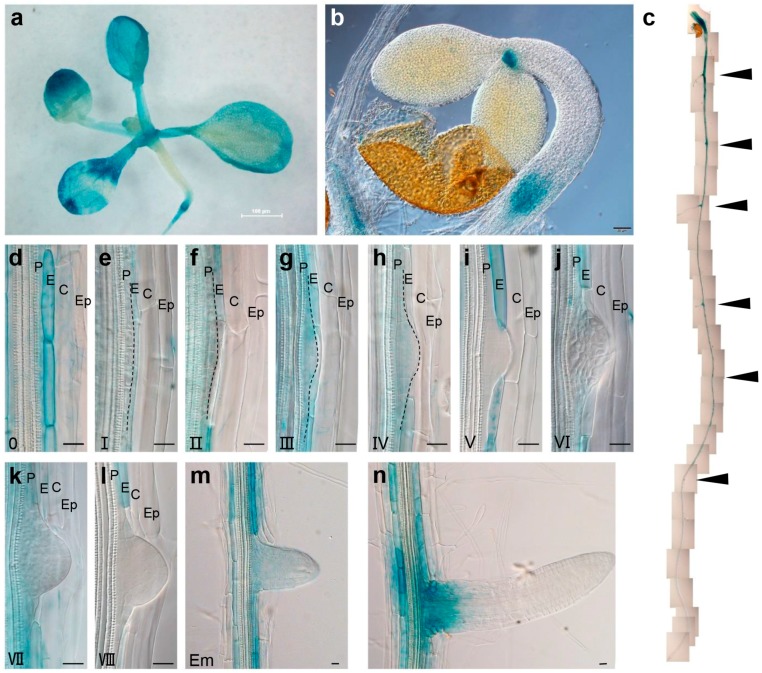
Expression pattern of *BRS1* in *Arabidopsis*. (**a**) GUS (β-glucuronidase) staining assay of *BRS1p-GUS* in the shoots of 12-day-old seedlings, bar = 100 μm; (**b**) 2-day-old seedlings, bar = 100 μm; (**c**) the whole root of 7-day-old seedlings; The arrow marked the LRs and LRPs; (**d**–**l**) A close-up view of the LRP in successive stages in 10-day-old seedlings, bar = 20 μm; (**m**,**n**) A view of the LRs following root staining. The black dotted line marks the LRP outline. P: pericycle; E: endodermis; C: cortex; Ep: epidermis. Bar = 20 μm.

Analysis using the GUS (β-glucuronidase) stain revealed that *BRS1* was strongly expressed in leaves (Figure 3a), shoot primordia (Figure 3b), and roots (Figure 3c), which was consistent with the previously reported tissue expression of *BRS1* [1,16]. However, *BRS1* was not evenly expressed in roots, with higher expression observed in distal roots compared with root tips, the division zone, and the elongation zone (Figure 3c), and especially high expression in the regions near to the LRs (indicated by the arrows in Figure 3c). Notably, the expression of *BRS1* showed clear cell type preference, with considerably higher expression in the endodermal cells of the parental root (Figure 3d–n) compared with the cortex, epidermis, and LRP.

### 2.4. BRS1 Localizes in the Extracellular Space around the LRP

**Figure 4:**
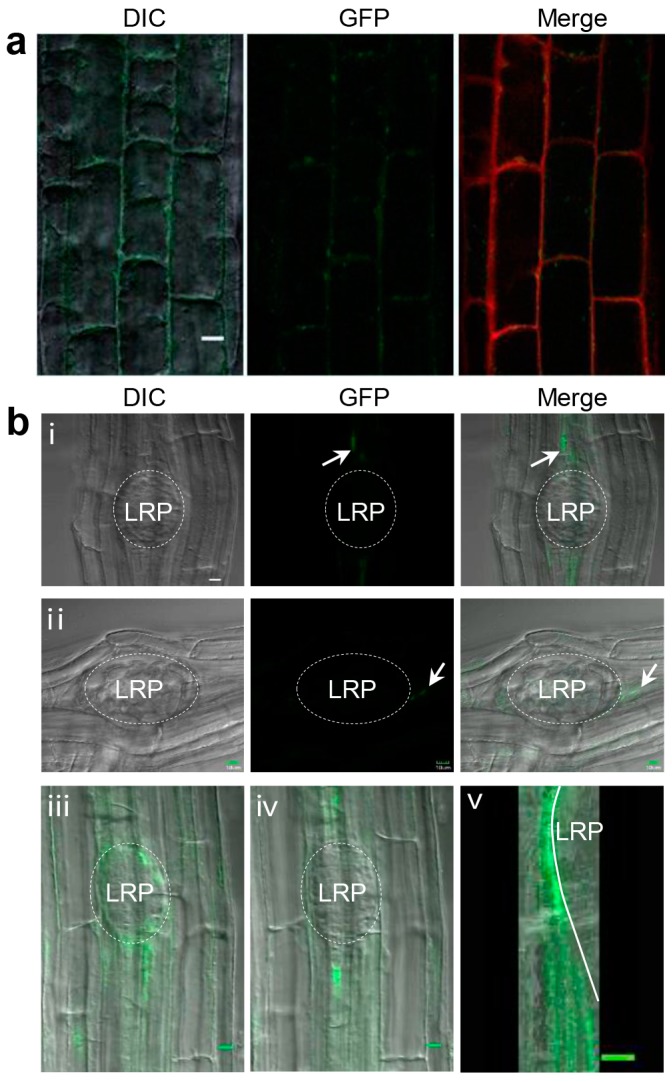
Subcellular localization of BRS1. (**a**) The subcellular distribution of *BRS1-GFP* was observed in the mature cells of the distal root region with propidium iodide stain for 5 min. The observations were performed on 12-day-old *35S-BRS1-GFP* seedlings. Bar = 10 μm; (**b**) The subcellular localization of BRS1 in the LRP of *35S-BRS1-GFP*; (**i**,**ii**) Front views of LRP; (**iii**,**iv**) The same LRP at different scanning layers; The arrow marked the position where BRS1 localization; (**v**) 48 images scanned continuously were compiled and this image stack was cut orthogonally (the scanning video is shown as a Supplementary Video). The white line marks the LRP outline. The roots were visualized using a confocal laser scanning microscope. Bar = 10 μm.

Previous studies have suggested that BRS1 is a secretory protein that localizes in the extracellular space [1,16]. In this study, the localization of BRS1 was further examined in the roots using *35S-BRS1-GFP*. BRS1 was found to localize in the extracellular space of mature root cells, overlapping with the cell wall (Figure 4a). Interestingly, BRS1 particularly accumulated at the interspace between the endodermal cells and cortex cells, which would separate to allow the LRs to pass through (Figure 4b) [26]. Further observations showed that BRS1 also strongly accumulated in the extracellular space around the LRP dome, when the LRP were passing through the endodermis and cortex cells (Figure 4b and the Supplementary Video), illustrating the main sites of action of BRS1.

### 2.5. Transcription of BRS1 Is Regulated by the Brassinosteroid (BR) Signaling

**Figure 5:**
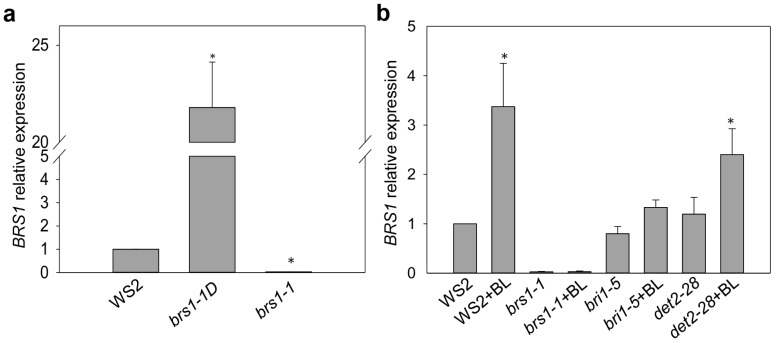
Relative *BRS1* transcript levels in root tissue. (**a**) *BRS1* transcript level in mutants; (**b**) *BRS1* transcript level in response to BL treatment. *BRS1* transcript levels were measured by qRT-PCR after 24-epi-brassinolide treatment. The data is shown for two independent biological replicates and three technical replicates ± SE. The asterisks indicate a significant difference from the corresponding control experiment by Student’s *t*-test (* *p* < 0.05).

A quantitative PCR was conducted to verify the over-expression status in *brs1-1D* in *Arabidopsis* root. Results showed that the transcription of BRS1 is up-regulated more than 20 times in *brs1-1D* compared to wild types (Figure 5a). To clarify the connection between the transcription of *BRS1* and BR signaling, we tested the *BRS1* response with brassinolide (BL) treatment in different genotypic background. In wild-type and *det2*, BR can dramatically induce the transcription of *BRS1*, but not remarkably in *bri1-5* (Figure 5b), indicating that BR positively regulates the transcription of *BRS1*, and this regulation is dependent on the BR signaling pathway. Unexpectedly, there was no significant difference in the transcriptional status among wild-type and BR mutants without BL treatment, indicating that the basic transcription of *BRS1* may be independent of BR homeostasis. These results demonstrated that *BRS1* was induced by the BR signaling in *Arabidopsis* root, and suggested that BR may facilitate the LPR emergence by inducing *BRS1*. 

## 3. Discussion

In this study, no obvious phenotypes were observed in two knockout mutants of BRS1 (in the WS and Col backgrounds, respectively), which was consistent with the high redundancy of BRS1 in *Arabidopsis* [1,15]. However, overexpressing *BRS1* resulted in an increase in LRs in different *Arabidopsis* ecotypes and BR mutants, and further observations clearly showed that BRS1 facilitates LR emergence.

BRS1 contains a signal peptide and localizes in the extracellular space [1,16]. As an SCP, BRS1 shows strong hydrolytic activity with a broad peptide substrate range in *Arabidopsis*, and its hydrolysis activity is necessary for its biological function [16]. Our results demonstrated a novel role of BRS1 in facilitating LR emergence, indicating that BRS1 may function in cell wall remodeling, similar to another extracellular localized SCP (NtSCP1) in tobacco, which operates via cell elongation [17]. LR emergence required multiple signallings for cell wall remodeling to separate cell contacts coordinately within the endodermis, cortex, and epidermis, and make way for LR emergence [28,30,34,36,38]. In this study, our results indicated BRS1 positively regulates the signaling of LR emergence. Previous observation also suggested BRS1 participates in BR signaling [1], and interferes with the signaling in *Arabidopsis* carpel development [15], indicating that BRS1 participates and/or disturbs plant signaling. Recent study suggested that SCP can trigger a peptide signal by acting on its peptide substrate [10], and regulated rice seed filling and germination process, suggesting a new signaling pathway that BRS1 may be involved in. Current study has demonstrated that peptides—the potential substrate of BRS1—in the root play a critical role in regulating root development as signaling molecules [39]. These findings indicated that BRS1 could be a promising regulator in peptide signaling to adjust plant growth and development. However, we could not rule out the other role of BRS1 via interaction with other components/pathways to regulate LR emergence. Therefore, the identification of BRS1 substrates or BRS1-interacting proteins would be critical for investigating the specific mechanism underlying BRS1 function.

The endodermis is located between the cortex and pericycle cells [40]. Unlike other root cells, the endodermal cells are surrounded by the rigid Casparian strip [25] and are regarded as the largest biomechanical obstacle to LR emergence [36]. Interestingly, BRS1 was highly expressed in root endodermal cells, possibly indicating that a major function of BRS1 may be in facilitating the LR breakthrough of the endodermis. The endodermis also functions as a root diffusion barrier because of the Casparian strip surrounding this cell layer that affects the directional control of water and solutes from both sides of the endodermis [25,40]. It is therefore critical that this region remains intact during LR emergence. The live observations revealed that BRS1 accumulated around the dome of the LRP (Supplementary Video), which may ensure the fine control of the gap between the LRP and endodermis. *BRS1* is expressed in the endodermis, but not in the LRP, indicating that LRP development is also controlled by the surrounding cells [36] and highlighting the unique role of the endodermis in LR emergence.

It should be noted that *35S-BRS1* show remarkably shorter primary roots as compensation for the increased number of LRs compared with *brs1-1D* (Figure 1a and Figure S1). The distinct root phenotypes might be explained by the fact that the native promoter of *BRS1* in the *brs1-1D* transgenic line remained intact [1], and *brs1-1D* therefore retained a functional promoter to constrain its expression in specific tissues and cells. Our observation revealed that native *BRS1* few expressed in root meristem and both root meristem development and root cell elongation were severely affected in *35S-BRS1* compared to *brs1-1D* (Figure S1), supporting that ectopic expressed *BRS1* disturb normal root structure formation and provide a potential strategy to optimize the shape of the root system by manipulating the expression of *BRS1*.

In conclusion, our observations suggested that BRS1 facilitates the passage of the LRP through the outer layer of cells, broadening the known functions of SCPs. Our findings also shed new light on the LR emergence process. Further identification of BRS1 substrates will provide insight into the underlying mechanisms involved.

## 4. Materials and Methods

### 4.1. Growth Conditions and Plant Materials

Wild-type (WT) Columbia (Col-0) and its mutants (*brs1-2*, *35S-BRS1-GFP* and *Col-BRS1p-GUS*), WT Wassilewskija (WS2) and its mutants (*brs1-1*, *brs1-1D* and *35S-BRS1*), as well as *bri1-5*, *bri1-5 35S-BRS1*, *bri1-5 brs1-1D*, *det2-28* and *det2-28 brs1-1D* mutants in a WS2 background were grown vertically on half-strength Murashige and Skoog (1/2 MS) plates at pH 5.6–5.8 (adjusted with 1 M KOH), supplemented with 0.85% (*w*/*v*) agar and 1% (*w*/*v*) sucrose. All plants were grown at 22 °C under long-day conditions (illumination intensity 100 µmol·m^−2^·s^−1^, 16 h light/8 h dark).

The *brs1-2* (SALK_114441) mutant was a T-DNA insertion mutant from the Salk Institute collection that had been verified by genotyping. *35S-BRS1-GFP* (Col), *35S-BRS1* (WS2), and *Col-BRS1p-GUS* were as previously described [16]; *bri1-5* is a weak mutant defective in the extracellular domain of BRI1, which is the receptor of brassinosteroids; *det2-28* is a BR biosynthetic mutant, which shows a significant reduction in BR biosynthesis; *bri1-5 brs1-1D*, an activation-tagging line with 4 × 35S enhancers inserted before the *BRS1* sequence, and *bri1-5 35S-BRS1* were as described previously and were verified by genotyping and RT-PCR [1,16]. The *WS2 brs1-1D* and *det2-28 brs1-1D* double mutants were generated by crossing WS2 and *det2-28* with *bri1-5 brs1-1D* respectively.

### 4.2. RNA Isolation and Quantitative Real-Time RT-PCR Analyses

Total RNA was extracted from the roots of eight-day-old WS2, *brs1-1* (WS2), *brs1-1D*, and *35S-BRS1* seedlings, using an RNeasy RNA plant extraction mini kit (Qiagen, Redwood City, CA, USA) according to the manufacturer’s protocol. One microgram of RNA was used for cDNA synthesis and quantitative real-time RT-PCR analysis.

Quantitative real-time RT-PCR was performed using a reagent kit (TaKaRa, Dalian, China) and 7300 Real-Time PCR System (Applied Biosystems, Foster City, CA, USA) and involved the use of a genomic DNA eraser, followed by reverse transcription and real-time PCR. The reaction was performed in a 20-µL volume in 96-well plates heated for 15 s at 95 °C for pre-denaturation, followed by 40 cycles of denaturation for 5 s at 95 °C and annealing for 31 s at 60 °C, and a final dissociation stage. Actin was included in the assay as a normalization control.

### 4.3. Histochemical β-Glucuronidase Assays

β-glucuronidase (GUS) staining of *Col BRS1p-GUS* transgenic plants was performed according to the following steps: firstly, plant tissues were fixed in 90% acetone on ice for 15 min, then the acetone was completely removed; secondly, the samples were immersed in GUS-solution: 2 mM X-Gluc, 5% (*v*/*v*) dimethylformamide, 10 mM EDTA-Na_2_ (pH 8.0), 0.1% (*v*/*v*) Triton X-100, 0.5 mM potassium ferricyanide (K_3_Fe(CN)_6_), 0.5 mM potassium ferrocyanide (K_4_Fe(CN)_6_), 50 mM phosphate buffered saline (pH 7.0), and vacuum dried for 15 min, followed by incubation at 37 °C for 11 h; finally, samples were successively washed in 100% and 75% alcohol.

### 4.4. Microscopic Analysis

Confocal laser scanning microscopy (Olympus IX83, Tokyo, Japan) was used to capture images of the GUS and green fluorescent protein (GFP) signals. For GFP signal detection, the argon laser excitation source was at 488 nm and the detection filters ranged from 505 to 525 nm. GUS-stained images were obtained and the LRP were rated using differential interference contrast (DIC) optics. To visualize the LRP phenotype clearly, GUS-stained roots were compressed in a tablet with transparent liquid consisting of 7.5% (*w*/*v*) gum arabic, 6 M chloral hydrate, and 5% (*v*/*v*) glycerine.

### 4.5. LRP Developmental Observations

Five-day-old seedlings grown on vertical 1/2 MS plates without sucrose [37] were turned 90° to stimulate the synchronous generation of the LRP at the bending site. All of the seedlings underwent gravitropic stimulation for 20 and 50 h (Col background), or 22 and 54 h (WS2 background), to analyze the developmental stages of induced LRP [21].

## Supplementary Materials

Supplementary materials can be found at www.mdpi.com/1422-0067/18/7/1549/s1.
