# Supplementary material for: BRS1 Function in Facilitating Lateral Root Emergence in Arabidopsis
Source: Int J Mol Sci. 2017 Jul 18;18(7):1549. doi: 10.3390/ijms18071549 (PMC5536037; doi:10.3390/ijms18071549)
Supplement: Supplementary file 1 [file ijms-18-01549-s001.zip › ijms-202463-supp.pdf]

BRS1 Function in Facilitating Lateral Root Emergence in *Arabidopsis*

Supplementary 1: Video. BRS1 accumulate at the dome of LPR. 48 continuously images were collected with microscopy (Olympus IX83) observation, and stacked using software ImageJ 1.48V.

Supplementary 2:

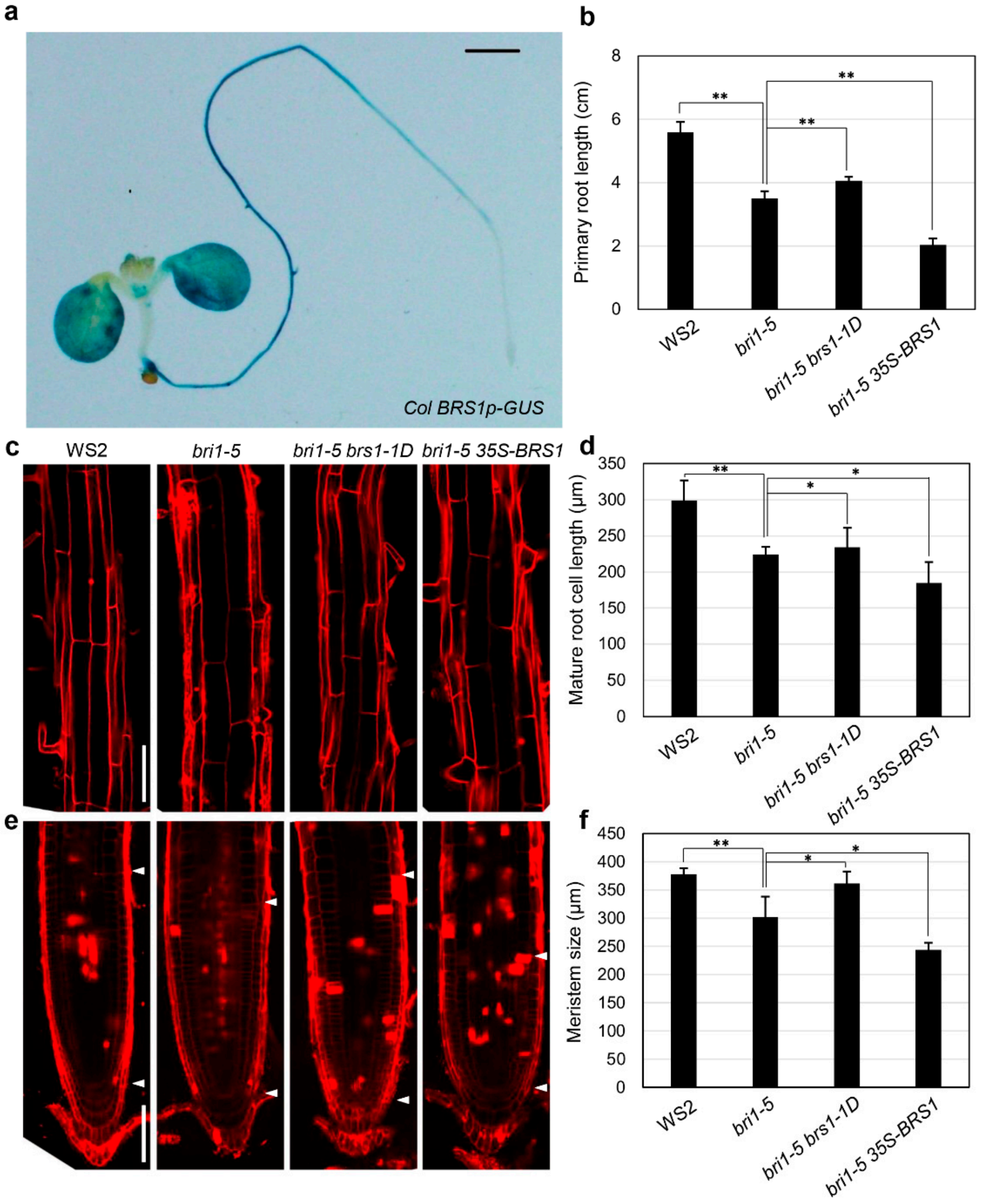

Figure S1. The variation of primary root length and primary root tip in different mutants and its WT (WS2). (**a**) GUS-staining assay of 9-day-old *BRS1p-GUS* transgenic line (Col background). Bar=1 mm. (**b**,**d**,**f**)The primary root length, mature root cell length and meristem size were quantified of WT (WS2), *bri1-5*, *bri1-5 brs1-1D* and *bri1-5 35S-BRS1* at 8 DAG. Mature root cell length were measured by root epidermis cell length in (**c**), and root meristem size were measured by distance between the elongation zone and the quiescent center, as marked by white triangles in (**e**). Data are presented as means ± SE (n > 15) from the independent experiments. The asterisks indicate a significant difference between compared lines with Student's t-test (* $p < 0.05$, ** $p < 0.01$). (**c**,**e**) Confocal fluorescence images of elongation zone cells and root tips in PI-stained primary root at 8 DAG. Bar = 100 μm.
